# Clinical-Genomic Risk Group Classification of Suspicious Lesions on Prostate Multiparametric-MRI

**DOI:** 10.3390/cancers15215240

**Published:** 2023-10-31

**Authors:** Radka Stoyanova, Olmo Zavala-Romero, Deukwoo Kwon, Adrian L. Breto, Isaac R. Xu, Ahmad Algohary, Mohammad Alhusseini, Sandra M. Gaston, Patricia Castillo, Oleksandr N. Kryvenko, Elai Davicioni, Bruno Nahar, Benjamin Spieler, Matthew C. Abramowitz, Alan Dal Pra, Dipen J. Parekh, Sanoj Punnen, Alan Pollack

**Affiliations:** 1 Department of Radiation Oncology, University of Miami Miller School of Medicine, Miami, FL 33136, USA; 2 Sylvester Comprehensive Cancer Center, University of Miami, Miami, FL 33136, USA; 3 Department of Public Health Sciences, University of Miami Miller School of Medicine, Miami, FL 33136, USA; 4 Department of Radiology, University of Miami Miller School of Medicine, Miami, FL 33136, USA; 5 Department of Pathology and Laboratory Medicine, University of Miami Miller School of Medicine, Miami, FL 33136, USA; 6 Desai Sethi Urology Institute, University of Miami Miller School of Medicine, Miami, FL 33136, USA; 7 Research and Development, Veracyte Inc., San Francisco, CA 94080, USA

**Keywords:** prostate cancer, multiparametric MRI, radiomics, Decipher, clinical-genomic risk classification

## Abstract

**Simple Summary:**

In this study, we built clinical- and radiomics-based models to predict lesions/patients at low risk based on a combined clinical-genomic classification system. Eighty-three multi-parametric MRI exams from 78 men were analyzed. Several models for lesion classification were built using a minimal clinical variables subset and radiomic features from the lesion and normal tissues. The models were also evaluated for patient classification. In all cases, the radiomic features improved the performance. To the best of our knowledge, this is the first study to demonstrate that machine learning radiomics-based models can predict patients’ risk using combined clinical-genomic classification.

**Abstract:**

The utilization of multi-parametric MRI (mpMRI) in clinical decisions regarding prostate cancer patients’ management has recently increased. After biopsy, clinicians can assess risk using National Comprehensive Cancer Network (NCCN) risk stratification schema and commercially available genomic classifiers, such as Decipher. We built radiomics-based models to predict lesions/patients at low risk prior to biopsy based on an established three-tier clinical-genomic classification system. Radiomic features were extracted from regions of positive biopsies and Normally Appearing Tissues (NAT) on T2-weighted and Diffusion-weighted Imaging. Using only clinical information available prior to biopsy, five models for predicting low-risk lesions/patients were evaluated, based on: 1: Clinical variables; 2: Lesion-based radiomic features; 3: Lesion and NAT radiomics; 4: Clinical and lesion-based radiomics; and 5: Clinical, lesion and NAT radiomic features. Eighty-three mpMRI exams from 78 men were analyzed. Models 1 and 2 performed similarly (Area under the receiver operating characteristic curve were 0.835 and 0.838, respectively), but radiomics significantly improved the lesion-based performance of the model in a subset analysis of patients with a negative Digital Rectal Exam (DRE). Adding normal tissue radiomics significantly improved the performance in all cases. Similar patterns were observed on patient-level models. To the best of our knowledge, this is the first study to demonstrate that machine learning radiomics-based models can predict patients’ risk using combined clinical-genomic classification.

## 1. Introduction

Prostate cancer is the most common cancer in American men. In 2022, more than a quarter of a million men was diagnosed with the disease [1]. Clinical decisions related to the choice of treatment, including active surveillance (AS), are multifactorial and complex. Prostate cancer risk assessment governs these decisions across a spectrum of local-regional diseases, from whether to biopsy to whether to intensify treatment using multimodality therapy. RNA transcript marker-based signatures have rapidly been incorporated into such determinations, along with Gleason Score (GS), now termed Grade Group (GG) [2]. While GG remains the standard of care, there has been a paradigm shift to incorporate transcriptomic signatures into clinical decision making. In particular, patients with GG1-3 cancer may be recommended to have either intensified or de-intensified treatment based on a genomic risk score [3,4].

Prostate needle biopsy carried out under multi-parametric MRI (mpMRI) guidance has gained acceptance as a key component of patient management. From the biopsy tissue, both histopathological and genomic biomarkers are used in clinical management. After prostate biopsy, clinicians have various tools for risk assessment, including the National Comprehensive Cancer Network (NCCN) [5] risk stratification schema. The primary goal of the NCCN risk assessment is to predict biochemical recurrence (BCR) rather than survival outcomes such as distant metastasis (DM). To improve the prediction of adverse events, the NCCN classifications were integrated into a three-tier classification system with a commercially available genomic classifier, Decipher (Veracyte Inc., San Francisco, CA, USA) [6], which was optimized to predict the risk of DM [7]. The resultant clinical-genomic classification system, referred to as the Spratt criteria, stratifies patients into low-, intermediate- and high-risk groups [7].

Active surveillance (AS) has emerged as a safe alternative to immediate treatment in low-risk patients [8,9,10]. AS has been incorporated into many prostate cancer management guidelines, which reduces the burden of overtreatment. While initially reserved for men with low-risk cancer (GS6 or GG1), there has been an increase in the inclusion of men with low-volume favorable intermediate-risk prostate cancer [11]. However, there is still a concern about missing the window for cure in men with greater than GG2 disease, as emerging data with long follow-up show an increased risk of metastasis [12]. Prostate cancer multifocality and heterogeneity [13] are the Achilles heel of prostate cancer risk stratification, and standard ultrasound template biopsies have proven to have poor Negative Predictive Value (NPV) for the detection of clinically significant prostate cancer [14]. We hypothesize that with improved techniques for tumor identification, targeting for biopsies, and classification using quantitative imaging, good candidates for AS would be reliably identified, including some with GG2 disease. We also hypothesize that patients in the low-risk group by the Spratt criteria will constitute patients who are good candidates for active surveillance. The low-risk classification is defined as either (i) low-risk based on NCCN and low- or intermediate-risk based on Decipher; or (ii) low-or-intermediate NCCN risk group and low Decipher group (Supplementary Figure S1).

The use of mpMRI for the detection and classification of prostate cancer is rapidly evolving due to its growing availability and the efforts in the radiology community to standardize the reporting of suspicious prostate lesions (Prostate Imaging Reporting and Data System (PI-RADS) [15] (current version PI-RADSv.2.1) [16]. Computer-aided diagnosis (CAD) techniques for quantitative mpMRI analysis have also been developed for prostate cancer detection and diagnosis [17,18,19,20,21,22]. The CAD efforts can be divided into two categories based on the main objectives for the analysis: (i) detection/segmentation of the suspicious lesion; and/or (ii) assessment of the aggressiveness of prostate cancer. The Habitat Risk Score (HRS) approach was developed to automatically identify suspicious lesions on mpMRI of the prostate and score the pixels within these regions by aggressiveness [23]. Advanced quantitative mpMRI features, also referred to as radiomic features, are extracted from the lesion volumes, and these variables are then used to build descriptive and predictive models [23].

In this manuscript, we combine clinical and radiomic features to develop a model to classify lesions and, consequently, patients prior to biopsy as low risk according to the Spratt criteria [7]. We utilize HRS to automatically segment on mpMRI the areas of the prostate biopsy and, using our radiomics pipeline [24], extract quantitative imaging features from the segmented region on multiple mpMRI sequences. Patients are classified as low risk if all mpMRI lesions are classified as low risk. To recreate a realistic scenario, only clinical features available a priori to biopsy are considered. The importance of the approach is that this model will allow non-invasive assessment prior to biopsy for patients who are good candidates for AS and may help delay biopsy or de-escalate surveillance biopsies.

## 2. Materials and Methods

### 2.1. Study Population

The study cohort comprised patients participating in two institutionally approved and registered trials: a single-arm active surveillance (AS) trial “MRI-Guided Biopsy Selection of Prostate Cancer Patients for Active Surveillance versus Treatment: The Miami MAST Trial“(ClinicalTrials.gov: NCT02242773) and a phase II randomized clinical trial “MRI-Guided Prostate Boosts Via Initial Lattice Stereotactic vs. Daily Moderately Hypofractionated Radiotherapy (BLaStM)” (ClinicalTrials.gov: NCT02307058). Both trials were approved by the Institutional Review Board at the University of Miami and all patients signed appropriate informed consent for treatment and the analysis of MRI and biopsy tissue for research purposes. Patients in both trials underwent mpMRI followed by MRI-ultrasound (MRI-US) fusion biopsies. Patients also agreed to have their tissue sent to Veracyte Inc. (San Francisco, CA, USA), and their data are included in this research. During the initial phase of the trials between 2014 and 2017, all cancer-positive biopsy cores with larger than 1 mm of cancer were sent to Veracyte for gene expression analysis. In addition, if available, positive cores from the patient’s diagnostic biopsy (prior to enrollment in the MAST/BLaStM clinical trials) were also sent.

### 2.2. Multiparametric-MRI of the Prostate

MRI sequences and sequence parameters were consistent with the recommendations for PI-RADSv2 [16]. The exams consisted of axial T2-weighted (T2W) MRI of the male pelvis, Diffusion-Weighted Imaging (DWI) with the generation of Apparent Diffusion Coefficient (ADC) maps and Dynamic Contrast-Enhanced (DCE)-MRI. mpMRI data was acquired using 3T Discovery MR750 (GE, Waukesha, WI, USA), 3T MR Magnetom Trio, Skyra and 1.5T Symphony (Siemens, Erlangen, Germany) magnets. Acquisition parameters of the individual sequences of mpMRI are given in Supplementary Table S1.

### 2.3. Workflow for Co-Registration of Genomic and Radiomic Data

**Figure 1:**
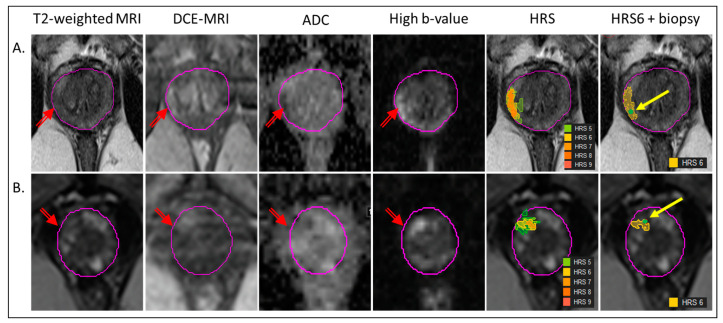
Co-registration of biopsy and segmentation of volumes for radiomics analysis. The radiogenomic pipeline is illustrated in the mpMRI from two patients (**A**,**B**). The lesion is marked with a red arrow on T2-weighted MRI, early enhancing Dynamic Contrast Enhancing (DCE)-MRI, Apparent Diffusion Coefficient (ADC) and High b-value (BVAL) image from the Diffusion-Weighted Imaging (DWI) sequence. Habitat Risk score (HRS) heat maps for HRS ≥ 5, associated with the lesion are overlaid on T2-weighted MRI. The last image illustrates the HRS6 volume in yellow, overlapping with the needle track (green dot, yellow arrow).

The image segmentation and the co-registration of the biopsy/gene expression and radiomics of the lesion are illustrated in Figure 1. Prostate and suspicious-for-cancer regions were outlined in Dynacad 5.1 (InVivo, Gainsville, FL, USA) by radiologists with more than ten years of experience in genitourinary (GU) malignancies using PI-RADSv2. The findings were also confirmed by heatmaps generated by HRS, described in detail in Stoyanova et al. [23]. Briefly, HRS is an approach that automatically assigns a score from 1 to 10 to each pixel in the prostate in an increasing fashion related to tumor aggressiveness. HRS combines quantitative characteristics from the diffusion and perfusion sequences of mpMRI and is displayed as a heat map overlaid on the T2-weighted images. HRS was developed in reference to prostatectomy GS and, in particular, HRS6, i.e., the volume comprised by pixels with HRS = 6, which were concordant with the tumor volumes from radical prostatectomy [23].

MRI-US biopsies were carried out in UroNav (InVivo, Gainsville, FL, USA). Tissue from the identified targets was obtained for pathology and gene expression analysis. For patients in BLaStM, only suspicious areas seen on mpMRI were sampled. For patients in MAST, standard template biopsies were also collected.

The biopsy needle track coordinates (beginning and end) were recorded and transferred in MIM 7.2.3 (MIM Software, Cleveland, OH, USA). The tumor Regions of Interest (ROIs) were assigned as the volumes of HRS = 6 coinciding with the individual needle tracks (Figure 1). In cases where the needle tracks were not recorded, the recorded location of the biopsy was used as a guide for selecting the biopsy ROIs. Two regions, representative of the normally appearing tissue peripheral zone PZ (NAPZ) and transition zone TZ (NATZ), were manually selected.

### 2.4. Normalization of T2W and BVAL Intensities

For normalization of the T2-weighted MRI intensities, a multireference normalization approach was utilized [25]. Using the “Region Growing Utility” in MIM, three reference contours were selected in the gluteus maximus (GM), femoral head, and bladder. The average intensity values from these contours were assigned a fixed reference value. For each patient, a spline function between the average and reference values was fitted. GM was the only anatomical structure that was consistently identified on high b-value images (BVAL) for all patients. BVAL images were normalized by GM.

### 2.5. Radiomic Analysis

Radiomic features were extracted as described in Kwon et al. [24] using a Java-based plugin in MIM. ROIs intensities (first-order radiomic features) on the three image modalities T2W, ADC and BVAL were characterized using nine histogram descriptors: 10%, 25%, 50%, 75%, 90%, mean, standard deviation, kurtosis, and skewness. Five texture (second-order radiomics) features: energy, entropy, correlation, homogeneity, and contrast, were extracted from T2W, ADC and BVAL using Haralick texture descriptors [26]. The features were calculated using the grey level co-occurrence matrices (GLCM) for each voxel underlying the contoured regions in the image. Voxel-wise texture measures were computed in 3D by sliding a window of size 5 × 5 × 5 across the image region enclosing the tumor volume. Image intensities were rescaled within a 0–255 range within the 5 × 5 × 5 window. The rationale for this local normalization, rather than global volume normalization, is that the objective was to obtain texture estimates in the normal-appearing tissues in addition to the tumor volumes. The GLCM was then computed in 3D using 128 bins in a 5 × 5 × 5 patch centered at each voxel [27]. The texture values for the whole tumor were then summarized using the voxel-wise textures. The nine histogram descriptors described above were calculated for each texture feature. The texture features were computed using C++ and the publicly available Insight ToolKit (ITK 5.2.1) software libraries for imaging (Kitware, Carrboro, NC, USA).

In summary, 162 quantitative imaging variables (Table 1) were analyzed: 3 modalities (T2W, ADC and BVAL) × 6 features (first-order: intensity (int) and second-order: energy (ene), entropy (ent), contrast(con), correlation (cor), and homogeneity (hom)) × 9 descriptors (10%, 25%, 50%, 75%, 90%, mean, standard deviation (SD), kurtosis (Kurt), and skewness (Skew)). In addition, 162 variables were extracted for NAPZ and NATZ, bringing the total analyzed radiomic features to 486. Here, and in the rest of the text, the imaging variables’ names are constructed by concatenating the abbreviations of the pertinent ROI (tumor, NATZ or NAPZ), image sequence, radiomics feature, and histogram descriptor (Table 1). For instance, L_ADC_int_50 refers to the 50% of the ADC intensity in the lesion (biopsy ROI).

### 2.6. Genomic Analysis

Tissue microdissection, RNA extraction, and amplification and microarray hybridization were performed in a Clinical Laboratory Improvement Amendments (CLIA)-certified laboratory facility, as described previously in [28]. Amplified products were fragmented and labeled using the Encore Biotin Module (NuGen, San Carlos, CA, USA) and hybridized to Human Exon 1.0 ST GeneChips (Affymetrix, Santa Clara, CA, USA). The Decipher Score is a 22-gene fixed signature or algorithm that ranges from 0.0 to 1.0, with higher scores related to an increased risk of prostate cancer metastasis. The test uses 0.45 and 0.6 as cutoff points to differentiate between low- versus intermediate- and intermediate- versus high-risk, respectively [29].

### 2.7. Calculation of Spratt Score

The Spratt clinical-genomic risk score is calculated by assigning a numeric value of 0, 1, 2 and 3 to the low, favorable intermediate, unfavorable intermediate, and high/very high NCCN risk groups, respectively [7], and a numeric value of 0, 1, or 2 to the low, intermediate, and high risk Decipher genomic classifier, respectively, and then adding these two values to obtain a clinical-genomic risk group (see Supplemental Figure S1). The Spratt Score can be reported as either a six-tier or a three-tier risk stratification, with higher scores indicating higher risk.

### 2.8. Modeling and Statistical Analysis

**Figure 2:**
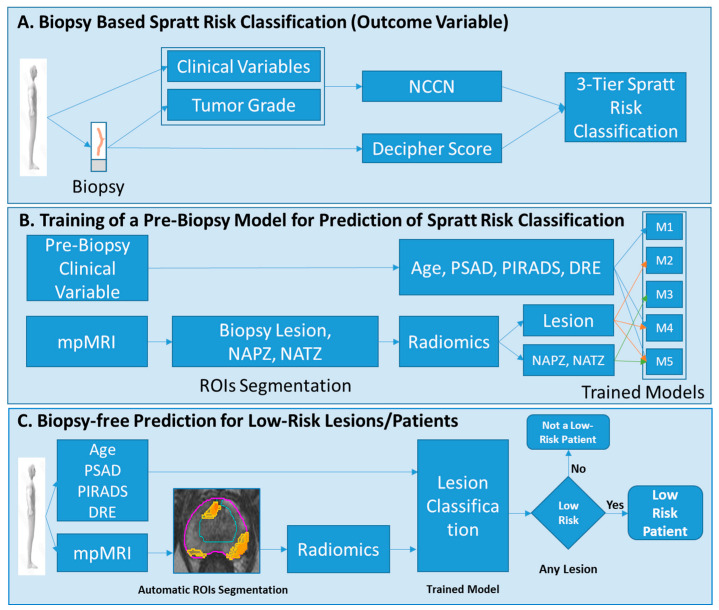
Modeling and analysis design. (**A**) Each biopsy is labeled using the Spratt criteria; (**B**) Five different models are trained, using the Spratt criteria’s classification from (**A**) to predict low-risk disease. The input parameters to the models are minimal clinical variables subset and radiomic features from the lesion, NAPZ and NATZ; (**C**) The developed models will be used to classify patients at low risk. Abbreviations: NCCN = National Comprehensive Cancer Network; NAPZ = normally appearing peripheral zone; NATZ = normally appearing transition zone; ROI = Region of Interest; PSA = Prostate Specific Antigen, PSAD = PSA density; DRE = digital rectal exam.

To evaluate the contribution of radiomic variables to the predictive value of the clinical variables, we evaluated five models for predicting low-risk lesions/patients as defined by Spratt’s criteria (Supplementary Figure S1). In Figure 2, the derivation of the outcome labels, model input variables and development, and the concept of future use are presented. The following models were generated:

Model 1: A clinical model using patient’s age, PSA density (PSAD), digital rectal exam (DRE), and PI-RADS for input. Importantly, this model did not incorporate the results of biopsy tissue, as we wanted to simulate a pre-biopsy clinical scenario and limited the variables to the initial prostate exam.

Model 2: Lesion-based radiomics model.

Model 3: Lesion and NAPZ/NATZ radiomics model.

Model 4: A combined clinical and lesion radiomics model.

Model 5: A combined clinical and lesion/NAPZ/NATZ radiomics model.

For Model 1, all pre-biopsy clinical variables were used. For the radiomics models, since imaging data are highly correlated and multi-dimensional, we used a penalized logistic regression model (GLMNET) to select important imaging variables that predict low-risk lesions [30]. Radiomics imaging data were standardized for feature selection. The volume of HRS6 (tumor volume) was added to all radiomics models. The variables were selected based on the adaptive LASSO method. Logistic regression analysis, using all clinical variables for Model 1 and the selected radiomic variables for Model 2 and Model 3, was used to predict low-risk lesions. For patient-level prediction, we selected the lesion with the highest three-tier score and evaluated the models. The performance of the models was also evaluated on a subset of patients that had negative DRE. The rationale for this subgroup analysis was to eliminate patients that most likely are not at low-risk.

The area under the receiver operating characteristic (ROC) curve (AUC) is reported as a performance measure for each trained model. AUC comparison is performed using the Venkatraman and Begg method [31]. For each model, bootstrap-based optimism-corrected AUC was calculated using 1000 runs [32]. Analysis was performed using corresponding R 4.2.3 packages (R Foundation for Statistical Computing, www.R-project.org (accessed on 1 May 2023)).

## 3. Results

A total of 231 biopsy cores from 78 men were analyzed for this study, with 46 men coming from the MAST active surveillance trial and 32 from the BlaStM primary radiation trial. (Table 2 and Table 3). As the patients in MAST were of low risk and in BlaStM of intermediate and high risk, the differences in the T-stage, PSA, DRE, GS/GG, Decipher, and the three-tier risk groups were statistically significant between the patients in the two trials.

A total of 83 mpMRI exams were analyzed. When available, both the diagnostic and protocol biopsies were matched with their corresponding mpMRIs. Thus, for five patients two mpMRI exams were analyzed. A detailed breakdown by MRI instruments is given in Table 2. The majority of the exams were acquired using Discovery, GE (50.6%), and Skyra, Siemens (39.8%) magnets.

In Table 4, the variables used for training each model are shown. All three pre-biopsy clinical variables were used in Model 1. Thirteen variables were selected for Model 2: Lesion Radiomics and 33 for Model 3: Lesion + NAPZ/NATZ Radiomics. The image intensity-based features (first-order radiomic features) were overrepresented (yellow highlight) in the lists of the significant variables; while they are one/sixth of all radiomic features, more than a third of the variables on both lists are related to image-intensities. For the lesion ROI, these variables are related to low T2 (L_t2_int_10) and high BVAL (L_b_int_75). In Supplementary Figure S2, four lesion imaging features used in the models: HRS6, L_t2_int_10, L_adc_int_50 and L_b_int_75 are displayed as box plots of the distribution of these features in low and intermediate/high-risk lesions. HRS6 (a surrogate of tumor volume) and BVAL were significantly higher, and T2 and ADC were significantly lower in intermediate/high risk in comparison with low-risk lesions.

**Figure 3:**
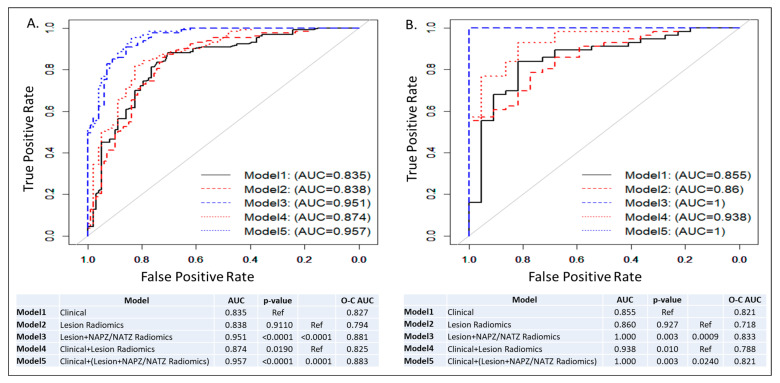
ROC curves and AUCs from five models: Model 1: Clinical model; Model 2: Lesion radiomics; Model 3: Lesion and NAPZ/NATZ radiomics; Model 4: Clinical variables with Lesion radiomics; Model 5: Clinical variables with Lesion and NAPZ/NATZ radiomics. (**A**) Lesion-based prediction for low risk, based on 231 lesions (133 low risk vs. 98 intermediate/high risk); (**B**) Patient-based prediction for low risk, based on 78 patients (56 low risk vs. 22 intermediate/high risk). The tables below the graphs show AUC, *p*-values and Optimism-Corrected (O-C) AUC.

**Figure 4:**
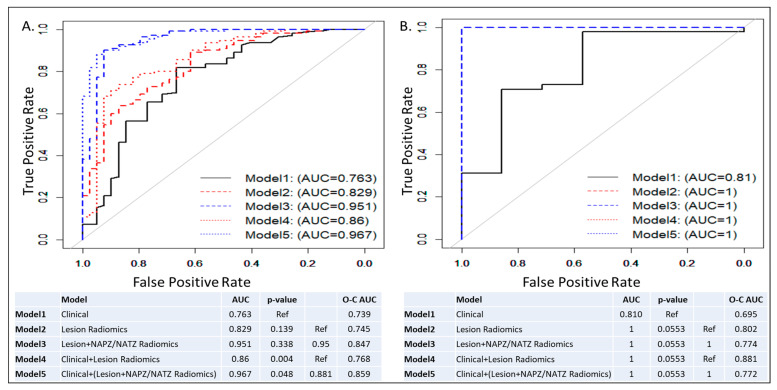
ROC curves and AUCs from five models in patients with negative DRE: Model 1: Clinical model; Model 2: Lesion radiomics; Model 3: Lesion and NAPZ/NATZ radiomics; Model 4: Clinical variables with Lesion radiomics; Model 5: Clinical variables with Lesion and NAPZ/NATZ radiomics. (**A**) Lesion-based prediction for low risk, based on 149 lesions (110 low risk vs. 39 intermediate/high risk); (**B**) Patient-based prediction for low risk, based on 55 patients (48 low risk vs. 7 intermediate/high risk). The tables below the graphs show AUC, *p*-values and Optimism-Corrected (O-C) AUC.

ROC curves and AUCs for the five trained models in all patients and subset analysis for patients with negative DRE (DRE = 0) are shown in Figure 3 and Figure 4. In each figure, the left panel shows the performance of the models on a biopsy level and on the right, it is shown on a patient level. In Figure 3, all lesions were analyzed: 133 low risk vs. 98 intermediate/high risk by the Spratt criteria. Interestingly, the clinical features performed similarly to the lesion radiomics. When the NAPZ/NATZ features were added to the model, there was a significant improvement in the performance, resulting in AUC of 0.95 for the best-performing Model 5. Similar patterns were observed on patient-level models (78 patients: 56 low-risk vs. 22 intermediate/high-risk). When only patients with DRE = 0 were considered, 149 lesions (110 low-risk vs. 39 intermediate/high risk) were analyzed (Figure 4). The prediction based on clinical variables degraded, indicating that positive DRE was a major driver in the performance in the previous models. The addition of the lesion radiomic features both in biopsy- and patient-level (55 patients: 48 low-risk vs. 7 intermediate/high-risk) significantly improved the classification.

Due to the high performance of the intensity’s features, we tested the value of using only first-order radiomics by re-creating models Model 1–5. There is a major advantage of using only intensities, assuming that the resultant model is not substantially underperforming relative to the full variable selection. Intensity futures are easy to compute, and they are highly reproducible between groups and software. The results from the intensities-only analysis are given in the Supplement. In Supplemental Table S2, the variables used for training each model are shown. All three clinical variables were used in Model 1. Six variables were selected for Model 2, and three of these variables overlapped with the ones selected in Table 4. In addition, L_t2_int_25 is strongly correlated with L_t2_int_10 (Table 4). Thirteen variables were selected for Model 3 and the lesion features were >50% of the total; from the normal-appearing tissues, only TZ features were selected. ROC curves and AUCs for the five trained models in all patients and subset analysis for patients with negative DRE (DRE = 0) are shown in Supplemental Figures S3 and S4. The overall performance for lesion-classification was in the range of 0.727 to 0.884 and the radiomic models degraded relatively to full-variable selection in all instances. The addition of the radiomic variables to the clinical improved the prediction only in Model 5 in the subset analysis.

## 4. Discussion

Image-based automated prostate cancer classification is an area of active investigation. The standard underlying schema of the approach consists of feature extraction and classification. In the majority of radiomic applications, the goal is to predict the Gleason Score/Group Grade (see recent review by Castillo et al. [33]). Here, we investigated the ability of a radiomic model to predict the low-risk group of patients based on a novel clinical-genomic classifier that is increasingly relevant to making clinical decisions. Such a model can prospectively improve patients’ selection for prostate biopsy and potentially tailor individual treatment decisions.

The main finding of our study is that it is feasible to build radiomics-based models that can predict the patient risk based on a clinical-genomic classifier. The performance of the lesion radiomics model (Model 2, AUC = 0.842) did not outperform the clinical Model 1, AUC = 0.8353. We hypothesized that the DRE results are driving the high performance of the clinical factors because of the strong association with abnormal DRE in high-risk patients. In a subsequent subset analysis, only of patients with negative findings on DRE (DRE = 0), Model 2’s AUC = 0.832 was markedly, albeit not significantly, higher than Model 1’s AUC = 0.763. In both cases, the AUCs of the lesion radiomic variables were almost the same, indicating the robustness of the model, while the exclusion of the DRE deteriorated the performance of the clinical variables. The combined models based on radiomics and clinical characteristics in all comparisons improved the predictive performance. Interestingly, radiomic features of the lesion environment, NAPZ and NATZ, contributed significantly to the overall model performance. In particular, the AUC of Model 1 (clinical variables) increased modestly, albeit significantly, from 0.835 to 0.871 in Model 4 (clinical + lesion radiomics). However, when the radiomics of PZ/TZ was added (Model 5), the AUC markedly increased to 0.962. The same pattern was observed in the subset analysis of patients with DRE = 0. Clinical decisions, on the other hand, are made on a per-patient basis rather than per-lesion. The outlined trends in model improvement after combining radiomic and clinical variables in Model 5 is also evident in per-patient models (Figure 3 and Figure 4, second panels).

As a topic of research interest, radiomics of prostate cancer has received increasing attention in recent years. The translation the identification of quantitative imaging features in the clinical workflow is the main driver for these developments. With this goal in mind, despite the promising results, these efforts largely have not been successful. One of the main challenges in our view is that the majority of the radiomic models are trained on predicating GS/GG. The histopathology cancer grade, although currently the best available predictor for disease outcome, is still a surrogate factor for the disease. Biopsies, and, hence GS/GG, are part of the clinical workflow; thus, an optimal radiomic model, fulfilling an unmet clinical need, should provide additional diagnostic/prognostic information. Finally, there is a serious problem with the reproducibility and generalizability of the radiomic features on images acquired under different acquisition sequence parameters and magnets.

To the best of our knowledge, this is the first report addressing the clinical decision-making related to biopsy for patients on active surveillance in the context of a clinical-genomic classifier. In realistic scenarios, we evaluated models, combining radiomics with the available clinical data after a mpMRI exam and before a biopsy. In Woznicki et al., a similar approach to the one used here for Model 4 was used to evaluate models based on clinical characteristics (PI-RADS, PSAD and DRE) and radiomics, and the reported results discriminated between (i) malignant vs. benign prostate lesion (AUC = 0.889) and (ii) clinically significant prostate cancer (csPCa) vs. clinically insignificant prostate cancer (cisPCa) (AUC = 0.844) [34] (csPCa is defined as GG grade 2 or higher [35]). They also conducted a subgroup analysis of patients with lesions <0.5 cc (of note, only the index lesion with the highest PI-RADS score or the biggest lesion volume was selected for analysis). The performance of the model for small lesions degraded: (i) malignant vs. benign prostate lesion (AUC = 0.678) and (ii) csPCa vs. cisPCa (AUC = 0.686). Here, we conducted a similar subgroup analysis but selected patients with negative DRE. DRE has low efficacy for prostate cancer detection with a pooled sensitivity of 0.51 and specificity of 0.59, as reported in a recent meta-analysis study [36]. However, a clearly abnormal DRE is highly associated with bulky and aggressive cancer and in the realistic scenario for selecting low-risk patients, patients with abnormal DRE will be excluded. In this sense, our subgroup analysis is more clinically relevant, as tumor volume is not readily available to be considered in patient management decisions.

The associations between prostate mpMRI features and gene expression have been investigated before [28]. We demonstrated that there are significant correlations between the quantitative imaging features and genes of three commercial signatures for prostate cancer assessment: Decipher^®^6, Prolaris^®^ Cell Cycle Progression (CCP) (Myriad Genetics, Salt Lake City, UT, USA) [37] and Genomic Prostate Score (GPS^®^) (Genomic Health, Redwood City, CA, USA) [38]. The presence of prostate cancer genomic prognostic signal in imaging was confirmed by Beksac et al. [39] and Hectors et al. [40]. Both studies identify individual sets of radiomic features that significantly correlate with GS and genomic signatures. Here, we report on models that predict clinical and genomic risk simultaneously.

Similar to our previous work [28], we analyzed multiple lesions per patient. This approach remains unique in the field, where typically only the index lesion is considered. In light of the genomic heterogeneity from different biopsy cores [13], the individual imaging characteristics of the biopsy locations need to be investigated in order to guide the prostate sampling to the highest-grade core with the highest genomic risk level.

Another unique aspect of our approach is the inclusion of NAPZ and NATZ in the radiomics pipeline. This was motivated by our previous gene ontology analysis that identified specific radiomic features, including from the tumor micro-environment, associated with immune/inflammatory response, metabolism, cell, and biological adhesion [28]. Indeed, as discussed above, when the radiomics of NAPZ/NATZ was considered (Model 5), the performance of the model significantly increased.

Despite the large heterogeneity in terms of magnets and manufacturers, about one-third of the variables on both lists are related to image intensities (Table 4, yellow highlight). For the tumor ROI, these variables are related to low T2 (L_t2_int_10) and BVAL (L_b_int_75). These characteristics: low T2-weighted and high intensities on the high b-value images, are used routinely by the radiologist in evaluating prostate mpMRI. As these radiomic variables are clearly related to the current practice for prostate mpMRI evaluation, we believe that this will increase the confidence of clinicians to use our models. We also developed models using only first-order radiomic features, and while the performance was satisfactory, it degraded significantly from the full variable selection setting.

Radiomic features were not extracted from the DCE sequence in mpMRI. One of the reasons is the high variability in DCE acquisitions and subsequent analysis. Computer-Aided Diagnosis (CAD) systems are hindered by an absence of standardization for DCE-MRI analysis. The Quantitative Imaging Network (QIN) at the National Cancer Institute (NCI) conducted two challenges among premier academic medical centers, and the evaluated pharmaco-kinetic parameters K^trans^, v_e_ and k_ep_ were quite variable [41,42]. However, the areas of relatively fast contrast wash-in and subsequent wash-out were utilized in the automatic tumor volume delineation (HRS6) [23]. In addition, there is a growing body of scientific articles reporting no performance difference for studies that included T2, diffusion-weighted imaging (DWI), and ADC images as compared to studies that added a DCE sequence. The DCE sequence is included in the PI-RADSv2; however, there is a debate about its added value [43]. In Monti et al. [44], radiomic models using T2W and ADC images performed better than an advanced model with additional diffusion kurtosis imaging and DCE for prostate cancer detection.

Our study has several limitations, including the relatively small sample size, precluding analysis of a separate validation set. Evaluation in a larger prospective cohort would be beneficial to validate these preliminary findings. This study was conducted within a single-institution academic setting with subspecialized multidisciplinary expertise and the performance of the created models may not be generalizable to community practice. In the analysis, we included only first- and second-order radiomic features to avoid overfitting.

## 5. Conclusions

In conclusion, our study demonstrated that a machine-learning radiomics-based model is capable of predicting novel clinical-genomic classifications. While there are several reports on the association of radiomic variables with GS/GG, to the best of our knowledge, this is the first report for predicting an integrated clinical-genomic classification.

## Figures and Tables

**Table 1:** Radiomic variables and the abbreviations used in radiomic variables name-convention *.

ROI	Image Sequence	Radiomics Feature	Histogram Descriptor
Lesion (L) Normal Appearing Peripheral Zone (NAPZ) Normal Appearing Transition Zone (NATZ)	T2-weighted (t2) ADC (adc) High b-value (b)	Intensity (int) Contrast (con) Correlation (cor) Energy (ene) Entropy (ent) Homogeneity (hom)	10% 25% 50% 75% 90% mean standard deviation (SD) kurtosis (Kurt) skewness (Skew)

Abbreviations: ROI = Region of Interest; ADC = Apparent Diffusion Coefficient. * Variable names are the concatenation of ROI, image sequence, radiomics feature and histogram descriptor for that feature. For example, the lesions’ ROI 90% energy texture on ADC will be L_adc_ene_90. Alternatively, NATZ_t2_cor_50 refers to the 50% of the correlation texture variable in NATZ on T2W MRI.

**Table 2:** Patient characteristics for study cohort organized by the clinical trial.

Variable	TOTAL (N (%))	MAST (N (%))	BLASTM (N (%))	*p*-Value ^a^
**Patients**	78	46	32	
**Age (median, range) years**	64.5 (44–82)	64.0 (44–82)	67.5 (44–79)	0.237
**Age groups**				0.559
≤44 years	2 (2.6)	1 (2.2)	1 (3.1)	
45 to 54 years	7 (9.0)	5 (10.9)	2 (6.3)	
55 to 64 years	30 (38.5)	20 (43.5)	10 (31.3)	
65 to 74 years	31 (39.7)	17 (37.0)	14 (43.8)	
≥75 years	8 (10.3)	3 (6.5)	5 (15.6)	
**Race/ethnicity**				0.218
Non-Hispanic White	37 (47.4)	25 (54.3)	12 (37.5)	
Non-Hispanic Black	11 (14.1)	6 (13.0)	5 (15.6)	
Hispanic/Latino	28 (35.9)	15 (32.6)	13 (40.6)	
Others	2 (2.6)	0 (0)	2 (6.3)	
**PSA, ng/mL, median, range**	6 (1.3–77.7)	4.7 (1.3–16.7)	9.7 (1.5–77.7)	0.0004
**PSA groups, ng/mL**				0.001
<10	57 (73.1)	40 (87.0)	17 (53.1)	
10–20	15 (19.2)	6 (13.0)	9 (21.8)	
>20	6 (7.7)	-	6 (18.8)	
**Grade Group**				<0.0001
1	40 (51.3)	36 (78.3)	4 (12.5)	
2	15 (19.2)	7 (15.2)	8 (25.0)	
3	10 (12.8)	3 (6.5)	7 (21.9)	
4–5	13 (16.7)	0 (0.0)	13 (40.6)	
**T stage**				<0.0001
T1	58 (74.4)	46 (100)	12 (37.5)	
T2–T3	20 (25.6)	0 (0)	20 (62.5)	
**N of biopsy**				0.3748
1	25 (32.1)	16 (34.8)	9 (28.1)	
2	10 (12.8)	7 (15.2)	3 (9.4)	
3	3 (25.6)	13 (28.3)	7 (21.9)	
4	9 (11.5)	5 (10.9)	4 (12.5)	
≥5	14 (17.9)	5 (10.9)	9 (28.1)	
**DRE ^b^**				<0.0001
0	55 (70.5)	46 (100)	9 (28.1)	
1	18 (23.1)	0 (0.0)	18 (56.3)	
2	5 (6.4)	0 (0.0)	5 (15.6)	
**PI-RADSv.2.1**				0.0002
1–2	28 (35.9)	22 (47.8)	6 (18.8)	
3	8 (10.3)	7 (15.2)	1 (3.1)	
4	23 (29.5)	12 (26.1)	11 (34.3)	
5	19 (24.3)	5 (10.9)	14 (43.8)	
**MRI scanner (Vendor)**				0.2029
3T Discovery (GE)	42 (50.6)	21 (42.0)	21 (63.6)	
3T Skyra (Siemens)	33 (39.8)	24 (48.0)	9 (27.3)	
3T TimTrio (Siemens)	6 (7.2)	4 (8.0)	2 (6.1)	
1.5T Symphony (Siemens)	2 (2.4)	1 (2.0)	1 (3.0)	

Abbreviations: PSA = Prostate Specific Antigen; DRE = Digital Rectal Exam; T = Tesla. ^a^ *p*-values from *t*-test for continuous variables or Fisher’s exact test for categorical variables; ^b^ DRE categories are: 0 = No palpable abnormality in the prostate or rectum; 1 = Palpable abnormality; 2 = Palpable abnormality, suggestive for extraprostatic extension.

**Table 3:** Biopsy characteristics for study cohort organized by clinical trial.

Variable	TOTAL (N (%))	MAST (N (%))	BLASTM (N (%))	*p*-Value ^a^
**Biopsy N**	231	124	107	
**Grade Group**				<0.0001
1	123 (53.2)	102 (82.3)	21 (19.6)	
2	46 (19.9)	18 (14.5)	28 (26.2)	
3	21 (9.1)	4 (3.2)	17 (15.9)	
4–5	41 (17.7)	-	41 (38.3)	
**Biopsy Type**				0.656
Diagnostic	75 (32.5)	23 (18.5)	52 (48.6)	
Trial	156 (67.5)	101 (81.5)	55 (51.4)	
**Decipher**				<0.0001
Low risk	161 (69.7)	114 (91.9)	47 (43.9)	
Intermediate risk	23 (10.0)	7 (5.7)	16 (15.0)	
High Risk	47 (20.3)	3 (2.4)	44 (41.1)	
**NCCN**				<0.0001
Group 1	90 (39.0)	79 (63.7)	11 (10.3)	
Group 2	48 (20.8)	28 (22.6)	20 (18.7)	
Group 3	41 (17.7)	14 (11.3)	27 (25.2)	
Group 4	52 (22.5)	3 (2.4)	49 (45.8)	
**3-tier classification system**				<0.0001
Low risk	133 (57.6)	103 (83.1)	30 (28.0)	
Intermediate risk	61 (26.4)	21 (16.9)	40 (37.4)	
High Risk	37 (16.0)	-	37 (34.6)	

Abbreviations: MAST = “MIAMI MRI selection for Active surveillance versus Treatment” Clinical Trial; BlaStM = “MRI-Guided Prostate Boosts Via Initial Lattice Stereotactic versus Daily Moderately Hypo-fractionated Radiotherapy” Phase II clinical Trial. ^a^ *p*-values from *t*-test for continuous variables or Fisher’s exact test for categorical variables.

**Table 4:** Variables for the three models for prediction. Variables in yellow are image-intensity related.

Clinical Variables Model 1	Lesion Radiomic Variables Model 2	Lesion/NAPZ/NATZ Radiomic Variables Model 3
Age (continuous) PSAD (continuous) DRE (0 vs. 1–2) PI-RADS (1–2 vs. 3,4,5)	HRS6 (volume) L_t2_int_10 L_adc_int_50 L_adc_int_Kur L_adc_int_Ske L_b_int_75 L_t2_con_25 L_adc_con_90 L_t2_ene_SD L_adc_ene_SD L_b_ene_25 L_b_ene_50 L_adc_ent_10 L_t2_hom_90	HRS6 (volume) L_t2_int_10 * L_adc_int_Ske * L_adc_con_90 * L_adc_cor_90 L_adc_ene_25 L_adc_ene_SD * L_b_ene_75 L_b_ent_10 L_t2_hom_90 * NATZ_t2_int_SD NATZ_t2_int_Ske NATZ_adc_int_90 NATZ_b_int_90 NATZ_b_int_SD NATZ_t2_con_SD NATZ_adc_con_10 NATZ_adc_cor_50 NATZ_t2_ene_SD NATZ_b_ene_75 NATZ_t2_ent_50 NATZ_b_hom_50 NAPZ_t2_int_75 NAPZ_t2_int_Kur NAPZ_t2_int_Ske NAPZ_b_int_90 NAPZ_b_int_Kur NAPZ_t2_con_75 NAPZ_adc_con_10 NAPZ_b_con_10 NAPZ_t2_cor_50 NAPZ_adc_cor_25 NAPZ_adc_ene_75 NAPZ_t2_ent_90 NAPZ_b_ent_10 NAPZ_t2_hom_90 NAPZ_t2_hom_SD NAPZ_b_hom_10 NAPZ_b_hom_SD

Abbreviations: PSAD = Prostate Specific Antigen density; DRE = Digital Rectal Exam; NAPZ = Normal Appearing Peripheral Zone; NATZ = Normal Appearing Transition Zone; HRS6 = Volume defined by pixels with Habitat Risk Score = 6. * Variables that are on both “Lesion” and “Lesion and NAPZ/NATZ” lists.

## Data Availability

Restrictions apply to the availability of these data. Data were obtained from [third party] and are available [from the authors] with the permission of [third party].

## Supplementary Materials

The following supporting information can be downloaded at: https://www.mdpi.com/article/10.3390/cancers15215240/s1, Figure S1: Clinic-genomic Classifier (Spratt criteria): Schema, modified from Spratt et al. [7], for combining National Comprehensive Cancer Network (NCCN) risk groups with Decipher groups to develop clinical-genomic point system, resulting in clinical-genomic 3-tier risk groups. The goal is to create clinical-radiomics model to predict lesions/patients at low risk. Figure S2: Significantly different quantitative imaging features in low vs intermediate/high risk lesions. Box and whisker plots comparing HRS6, T2-weighted, ADC, and high B-value features in low and intermediate/high risk. Abbreviations: a.u. = arbitrary units. Figure S3: ROC curves and AUCs from five models using intensities features only: Model 1: Clinical model; Model 2: Lesion radiomics; Model 3: Lesion and NAPZ/NATZ radiomics; Model 4: Clinical variables with Lesion radiomics; Model 5: Clinical variables with Lesion and NAPZ/NATZ radiomics. (A) Lesion-based prediction for low risk, based on 231 lesions (133 low risk vs 98 intermediate/high risk); (B) Patient-based prediction for low risk, based on 78 patients (56 low risk vs 22 intermediate/high risk). The tables below the graphs show AUC, *p*-values and Optimism-Corrected (O-C) AUC. Figure S4: ROC curves and AUCs from five models using intensities features only in patients with negative DRE: Model 1: Clinical model; Model 2: Lesion radiomics; Model 3: Lesion and NAPZ/NATZ radiomics; Model 4: Clinical variables with Lesion radiomics; Model 5: Clinical variables with Lesion and NAPZ/NATZ radiomics. (A) Lesion-based prediction for low risk, based on 149 lesions (110 low risk vs 39 intermediate/high risk); (B) Patient-based prediction for low risk, based on 55 patients (48 low risk vs 7 intermediate/high risk). The tables below the graphs show AUC, *p*-values and Optimism-Corrected (O-C) AUC. Table S1: MRI acquisition parameters for mpMRI sequences. Table S2: Variables for the three models using only intensity-based radiomics features for prediction.
