# Supplementary material for: Clinical-Genomic Risk Group Classification of Suspicious Lesions on Prostate Multiparametric-MRI
Source: Cancers (Basel). 2023 Oct 31;15(21):5240. doi: 10.3390/cancers15215240 (PMC10647832; doi:10.3390/cancers15215240)
Supplement: Supplementary file 1 [file cancers-15-05240-s001.zip › cancers-2641288-SI.pdf]

cancers

MDPI

Supplementary Materials

Clinical-Genomic Risk Group Classification of Suspicious Lesions on Prostate Multiparametric-MRI

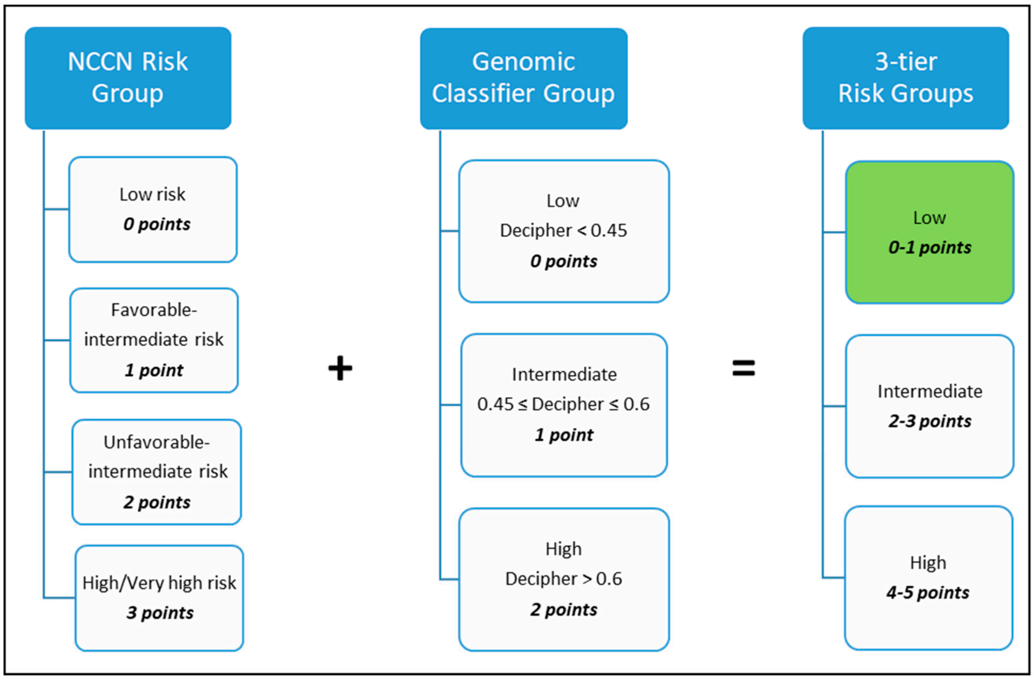

Supplemental Figure S1: Clinic-genomic Classifier (Spratt criteria): Schema, modified from Spratt et al [7], for combining National Comprehensive Cancer Network (NCCN) risk groups with Decipher groups to develop clinical-genomic point system, resulting in clinical-genomic 3-tier risk groups. The goal is to create clinical-radiomics model to predict lesions/patients at low risk.

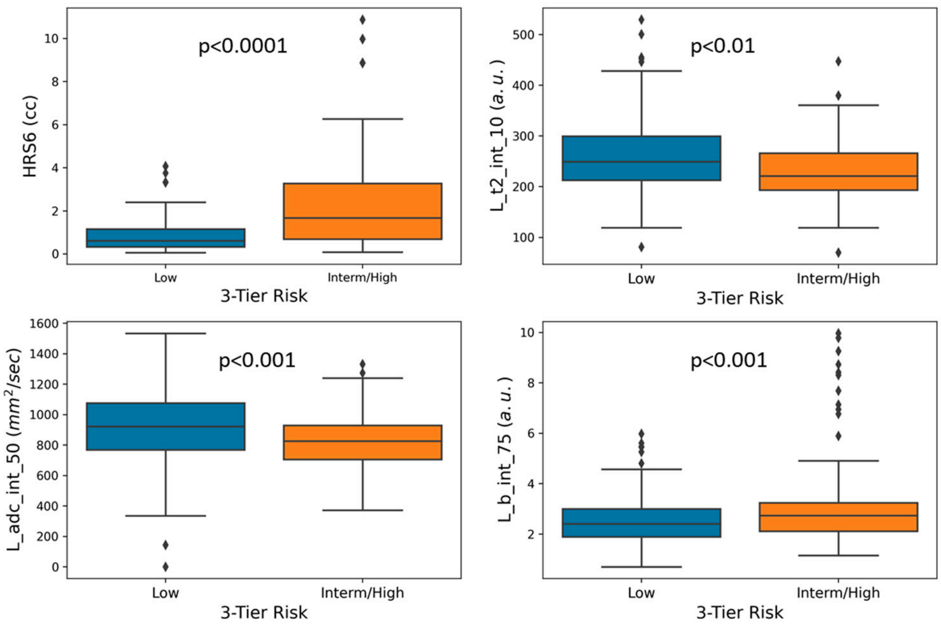

Supplemental Figure S2: Significantly different quantitative imaging features in low vs intermediate/high risk lesions. Box and whisker plots comparing HRS6, T2-weighted, ADC, and high B-value features in low and intermediate/high risk. Abbreviations: a.u. = arbitrary units.

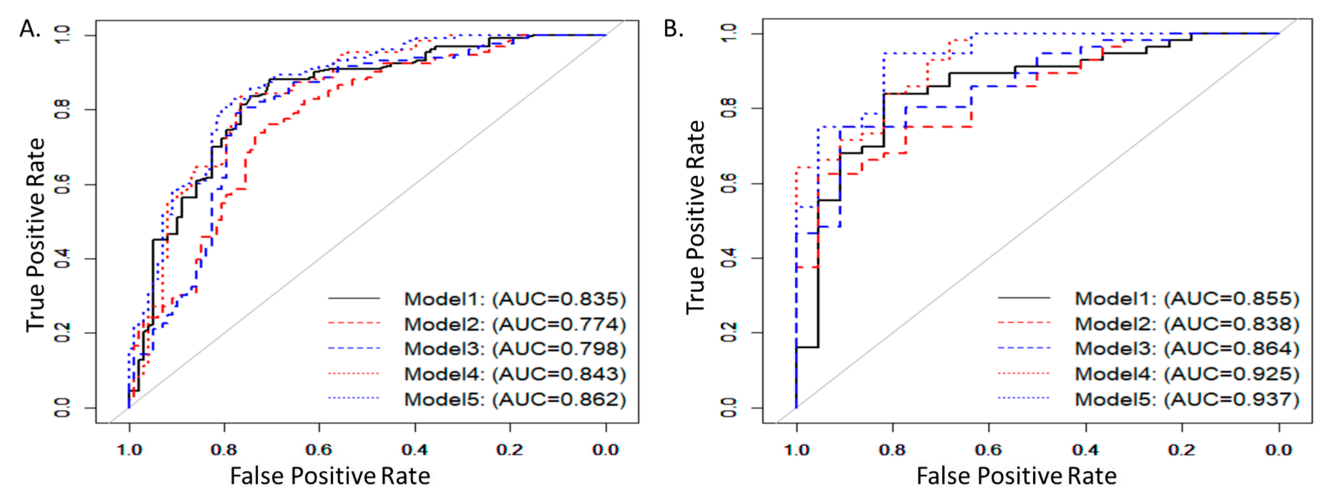

A.

	Model	AUC	p-value		OC-AUC
**Model1**	Clinical	0.835	Ref		0.827
**Model2**	Lesion Radiomics	0.774	0.044	Ref	0.746
**Model3**	Lesion+NAPZ/NATZ Radiomics	0.798	0.247	0.160	0.750
**Model4**	Clinical+Lesion Radiomics	0.843	0.511	Ref	0.811
**Model5**	Clinical+(Lesion+NAPZ/NATZ Radiomics)	0.862	0.145	0.150	0.812

B.

	Model	AUC	p-value		OC-AUC
**Model1**	Clinical	0.855	Ref		0.82
**Model2**	Lesion Radiomics	0.838	0.778	Ref	0.767
**Model3**	Lesion+NAPZ/NATZ Radiomics	0.864	0.870	0.411	0.727
**Model4**	Clinical+Lesion Radiomics	0.925	0.038	Ref	0.844
**Model5**	Clinical+(Lesion+NAPZ/NATZ Radiomics)	0.937	0.080	0.585	0.784

Supplemental Figure S3: ROC curves and AUCs from five models using intensities features only: Model 1: Clinical model; Model 2: Lesion radiomics; Model 3: Lesion and NAPZ/NATZ radiomics; Model 4: Clinical variables with Lesion radiomics; Model 5: Clinical variables with Lesion and NAPZ/NATZ radiomics. (A) Lesion-based prediction for low risk, based on 231 lesions (133 low risk vs 98 intermediate/high risk); (B) Patient-based prediction for low risk, based on 78 patients (56 low risk vs 22 intermediate/high risk). The tables below the graphs show AUC, p-values and Optimism-Corrected (O-C) AUC.

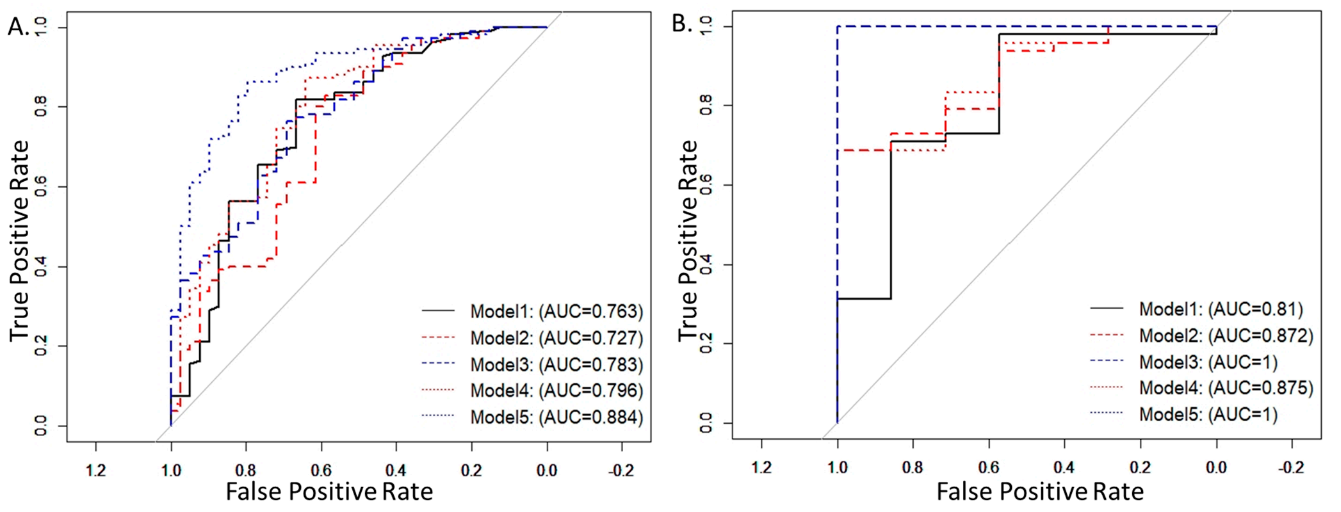

A.

	Model	AUC	p-value		OC-AUC
**Model1**	Clinical	0.763	Ref		0.827
**Model2**	Lesion Radiomics	0.727	0.446	Ref	0.794
**Model3**	Lesion+NAPZ/NATZ Radiomics	0.783	0.742	0.143	0.881
**Model4**	Clinical+Lesion Radiomics	0.796	0.154	Ref	0.825
**Model5**	Clinical+(Lesion+NAPZ/NATZ Radiomics)	0.884	0.003	0.004	0.883

B.

	Model	AUC	p-value		OC-AUC
**Model1**	Clinical	0.81	ref		0.821
**Model2**	Lesion Radiomics	0.872	0.499	Ref	0.718
**Model3**	Lesion+NAPZ/NATZ Radiomics	1	0.055	-	0.803
**Model4**	Clinical+Lesion Radiomics	0.875	0.465	Ref	0.788
**Model5**	Clinical+(Lesion+NAPZ/NATZ Radiomics)	1	0.055	-	0.821

Supplemental Figure S4: ROC curves and AUCs from five models using intensities features only in patients with negative DRE: Model 1: Clinical model; Model 2: Lesion radiomics; Model 3: Lesion and NAPZ/NATZ radiomics; Model 4: Clinical variables with Lesion radiomics; Model 5: Clinical variables with Lesion and NAPZ/NATZ radiomics. (A) Lesion-based prediction for low risk, based on 149 lesions (110 low risk vs 39 intermediate/high risk); (B) Patient-based prediction for low risk, based on 55 patients (48 low risk vs 7 intermediate/high risk). The tables below the graphs show AUC, p-values and Optimism-Corrected (O-C) AUC.

Supplementary Table S1: MRI acquisition parameters for mpMRI sequences.

Scanner	Parameters	T2W	DWI	DCE
**GE-Discovery**	Pulse Sequence	FSE	EPI	SPRG
	TR (ms)	10763	9500	4.052
	TE (ms)	104.944	52.6	1.78
	Pixel size (mm)	1.25 × 1.25 × 2.5	1.25 × 1.25 × 2.5	1.25 × 1.25 × 2.5
	Matrix	256 × 256 × 72	256 × 256 × 36	256 × 256 × 72
	b-values		50 – 500 – 1000	
	DCE-MRI Temporal resolution (sec)			27-36
**Siemens-Skyra**	Pulse Sequence	FSE	EPI	GR SP
	TR (ms)	6100	6600	5.24
	TE (ms)	114	91	2.33
	Pixel size (mm)	0.7 × 0.7 × 2.5	2.93 × 2.93 × 2.5	0.7 × 0.7 × 2.5
	Matrix	512 × 384 × 72	128 × 96 × 38	512 × 384 × 72
	b-values		50 – 500 – 1400	
	DCE-MRI Temporal resolution (sec)			30-35

Abbreviations: T2W = T2-weighted MRI; DWI = Diffusion-Weighted Imaging; DCE = Dynamic Contrast-Enhanced MRI.

Supplemental Table S2: Variables for the three models using only intensity-based radiomics features for prediction.

Clinical Variables	Lesion Radiomics Variables (*Intensity-based futures ONLY)*	Lesion/NAPZ/NATZ Radiomics Variables (*Intensity-based futures ONLY)*
Model 1	*Model 2*	*Model 3*
Age (continuous)<br>PSAD (continuous)<br>DRE (0 vs 1-2)<br>PI-RADS (1-2 vs 3,4,5)	HRS6 (volume)<br>L_t2_int_25<br>L_t2_int_Ske<br>L_adc_int_50<br>L_adc_int_Kur<br>L_b_int_75<br>L_b_int_SD	HRS6 (volume)<br>L_t2_int_25*<br>L_t2_int_SD<br>L_t2_int_Ske*<br>L_adc_int_50*<br>L_adc_int_Kur*<br>L_b_int_75*<br>L_b_int_Kur<br>NATZ_t2_int_10<br>NATZ_t2_int_50<br>NATZ_t2_int_Ske<br>NATZ_adc_int_90<br>NATZ_adc_int_Ske<br>NATZ_b_int_Kur

Abbreviations: PSAD = Prostate Specific Antigen Density; DRE = Digital Rectal Exam; NAPZ = Normal Appearing Peripheral Zone; NATZ = Normal Appearing Transition Zone; HRS6 = Volume defined by pixels with Habitat Risk Score = 6.
*Variables that are on both "Lesion" and "Lesion and NAPZ/NATZ list.
